# Q&A: targeting autophagy in cancer—a new therapeutic?

**DOI:** 10.1186/2049-3002-2-14

**Published:** 2014-09-11

**Authors:** Eileen White

**Affiliations:** 1 Rutgers Cancer Institute of New Jersey, Rutgers University, New Brunswick, NJ 08903, USA

**Keywords:** Autophagy, Cancer, Metabolism, Lysosome, KRAS, BRAF

## Abstract

Macroautophagy (autophagy hereafter) captures and degrades intracellular proteins and organelles in lysosomes as a quality control mechanism and recycles their components to sustain survival in starvation. Cellular self-cannibalization by autophagy is thought to have a context-dependent role in cancer. Autophagy inactivation is destructive to normal tissues and can promote cancer initiation while some established cancers upregulate autophagy that promotes their survival. We are only beginning to understand the role of autophagy in cancer and the precise mechanisms behind tumour suppression and promotion and the molecular and physiological contexts involved.

## Opinion

### Autophagy has emerged as a fundamental process in cell biology, meaning ‘to eat oneself’. What is autophagy and how was it discovered?

It was discovered by the Ohsumi lab in Japan as a pathway essential for yeast to survive nitrogen starvation. They discovered that yeast cells cannibalised their intercellular proteins and organelles and degraded them in the yeast lysosome—the vacuole. Many laboratories proceeded to investigate the reason why autophagy was important, and it is now clear that autophagy is essential for removing damaged intracellular components, by taking away toxic garbage to maintain homeostasis. But the other function is to take that degraded garbage and recycle the building blocks to sustain metabolism in starvation.

### Autophagy has in the past been described as a type of cell death, along with apoptosis and necrosis. But as you say, it is actually a method of survival—of prolonging cell viability when nutrients are low. Can you explain this apparent paradox?

The concept of autophagic cell death arose from the fact that, in some dying cells, there were lots and lots of autophagosomes. That led the observers to think that the presence of autophagosomes meant that autophagy was responsible for the death of those cells. Since that time—and this is long ago—we have come to realise that cell death is often associated with autophagy but is not caused by autophagy. In fact, in most cases, it is probably the cell inducing autophagy as an attempt to save itself, in response to a particular stress. Progressive, unrelenting autophagy, however, could potentially be a means to cell death.

So autophagy is clearly linked to cell survival. The most clear example is the one in yeast that I provided. There is also some data that if you knock out the autophagy pathway in mouse embryogenesis, the mouse will be born but will fail to survive neonatal starvation. We have data that if you turn off autophagy in an adult mouse, the mouse is fine for a while but is intolerant to starvation, and over time, it will have deterioration of a number of key tissues like the liver, brain and muscle [1]. So, autophagy is a critical homeostatic mechanism as it promotes tissue health and survival, and this is a function conserved from yeast to mammals.

### You mentioned how autophagy promotes tissue survival. You have spent a lot of time researching cancer and the role for autophagy there. Can you talk about how cancer cells use autophagy to survive?

We worked in the apoptosis field for many, many years and we accidentally stumbled upon autophagy when we were looking at cancer cells where the cell death pathway of apoptosis was shut off. What we found was that these cancer cells could survive prolonged starvation. That made no sense because all we had done was turn off the cell death mechanism. When we looked at these cells closely, we found out that they had activated the autophagy pathway and were surviving by autophagy. That was clear because if we deleted autophagy in these tumour cells and then starved them, then they now died.

That revealed to us for the first time that autophagy was a survival pathway that normal cells use, which cancer cells could take advantage as well. We felt very strongly that if there was a survival pathway used by cancer cells, we need to inhibit it to promote the demise of those cancer cells. At that point, my lab transitioned from working on mostly apoptosis to working on mostly autophagy. Shortly thereafter we looked into tumours and discovered that autophagy was often upregulated in hypoxic tumour regions—those are regions deprived of oxygen—and when we delete autophagy in those tumour cells, then you end up with no tumour cells surviving hypoxia. So, that suggested that even *in vivo*, autophagy was an important survival mechanism for cancer cells.

### Given that cancer cells can be reliant on autophagy, is there a way that we can use this reliance to develop new treatments for cancer?

That is the whole idea. But that is a very big job and a very big commitment, and so we felt that what we needed to do was to use the most powerful and physiological tools at our disposal to validate that autophagy was in fact a critical survival mechanism for certain cancers and try to figure out which cancers those are.

We embarked on large projects where we deleted an essential autophagy gene in genetically engineered mouse models for cancer. In these mouse models, cancers arise through activation of a specific oncogene and/or deletion of a known tumour suppressor gene, very much like what would happen naturally in a human. The tumours evolve from these events in a single cell, and with a functioning normal immune system. When you inactivate autophagy in *k-ras*- and *b-raf*-driven models of non-small-cell lung cancer, there is a dramatic reduction in tumour growth. That encouraged us that, yes, in the most physiological settings we can model in the laboratory, at least in those models for lung cancer, autophagy was providing an important survival function for those cancers.

I think it is worthwhile now to consider targeting some of the key components of the autophagy pathway with small molecules to try to attempt to block autophagy in patients. There are, however, clinical trials that are currently active using hydroxychloroquine, which is a lysosomotropic drug that interferes with the function of lysosome. When cells eat themselves by autophagy, ultimately all the cargo gets degraded in the lysosome. Hydroxycholoroquine is a currently available drug for malaria prophylaxis that can block the degradation of the cargo delivered to the lysosome by autophagy. Recent papers have just been published that suggest that there might be some activity of hydroxychloroquine in various human clinical trials for cancer.

### Given that autophagy is an essential process for normal cells, is there a risk that blocking autophagy would have some toxicity?

Absolutely an important question. Once we had evidence that shutting off autophagy in a tumour impaired the growth of those tumours, we then designed mouse models to test exactly that. This involved making a mouse where we can shut off autophagy by deleting an essential autophagy gene at will throughout the mouse, by providing a small drug—in this case tamoxifen—that can control the deletion of an essential autophagy gene. We engineered those mice so that they would also be separately able to create lung cancer when we had the mice inhale an adenovirus expressing Cre recombinase to activate RAS and to delete p53 in the lung [1].

The bottom line is that we could shut autophagy off in an adult mouse and then look to see how the normal adult mouse responded—that was step number one. Then, we would have an idea of what the toxicity would be of an autophagy inhibitor. Then, in step two, we could make a mouse with lung cancer and then delete the essential autophagy gene throughout the mouse, mimicking cancer therapy, and we could see if the tumour died before the deleterious consequences to the normal tissues of the mouse.

The results from those experiments are that when we take an adult mouse and we delete an autophagy gene throughout the whole mouse, the mouse is basically fine for a while [1]. This was good news; our data showed that normal tissues could, at least in the short term, tolerate the absence of autophagy. Eventually, at between 2 and 3 months post-deletion, the mice showed damage to important tissues like the liver, brain and muscle and died [1]. So there is a window where loss of autophagy has not too deleterious consequences to the mouse.

With that good news, we made mice with lung cancer and then we shut autophagy off. We looked 5 weeks after autophagy was ablated systemically in the mice and the normal tissues at that point are mostly fine, but the tumours were destroyed [1]. That was, I think, the genetic experiment that establishes that, at least in lung cancer, systemic ablation of autophagy may be therapeutically valuable.

### Will autophagy inhibition work as a treatment for many different cancers, or just to some?

I think it is entirely possible that not all tumours will be sensitive to autophagy inhibition. Right now, we do not really have a way to tell, although a number of people have ideas. I think we have an evidence that lung cancer is a good place to start. We are making other mouse models, for example prostate cancer and melanoma, using activation of different oncogenes and deficiency in different tumour suppressor genes, so that the mouse models might direct us to one cancer over another, depending on what the results are.

The pharmaceutical industry is working on autophagy inhibitors, so it would be very nice to have those reagents to test in the laboratory to see how effective they are. You have to remember that our genetic experiments represent an extreme case; we have complete loss of an essential autophagy gene and it is irreversible. So with a small-molecule autophagy inhibitor, you are not likely to get 100% inhibition that is irreversible. That is good and that is bad. It may be that it will be bad if we cannot inhibit autophagy efficiently enough, but on the other hand, it might be good because you may not need to inhibit autophagy completely for five consecutive weeks for the deleterious effects to appear in tumours. If it is possible to have 90% inhibition of the pathway for a shorter period of time, and normal tissue can recover faster than tumour tissue, it might be very valuable.

## Competing interests

Dr. White is a member of the Scientific Advisory Board of Forma Therapeutics.

## Author information

Eileen White, Ph.D., is Associate Director for Basic Science at the Rutgers Cancer Institute of New Jersey in New Brunswick, NJ. She talks to *Cancer & Metabolism* about autophagy, its role in cancer and the development of new therapeutics.
